# Prevalence and Factors Associated with Psychological Problems of Healthcare Workforce in Vietnam: Findings from COVID-19 Hotspots in the National Second Wave

**DOI:** 10.3390/healthcare9060718

**Published:** 2021-06-11

**Authors:** Nguyen Quang Tuan, Nguyen Doan Phuong, Dao Xuan Co, Do Ngoc Son, Luong Quoc Chinh, Nguyen Huu Dung, Pham The Thach, Nguyen Quoc Thai, Truong Anh Thu, Nguyen Anh Tuan, Bui Van San, Vu Son Tung, Ngo Van An, Do Nam Khanh, Vo Hoang Long, Nguyen Tai, To Muoi, Nguyen Dai Vinh, Nguyen Trong Thien, Le Duc Nhan, Nguyen Van Tuan

**Affiliations:** 1 Bach Mai Hospital, Hanoi 100000, Vietnam; quangtuanvtm@gmail.com (N.Q.T.); daoxuanco@gmail.com (D.X.C.); 2 National Institute of Mental Health, Bach Mai Hospital, Hanoi 100000, Vietnam; doanphuong@gmail.com (N.D.P.); buivansan@hmu.edu.vn (B.V.S.); Vusontung269@gmail.com (V.S.T.); 3 Center for Emergency Medicine, Bach Mai Hospital, Hanoi 100000, Vietnam; sonngocdo@gmail.com (D.N.S.); luongquocchinh@gmail.com (L.Q.C.); bstuanccbm@gmail.com (N.A.T.); 4 Nephro-Urology and Dialysis Center, Bach Mai Hospital, Hanoi 100000, Vietnam; nhdungbm@gmail.com; 5 Department of Intensive Care, Bach Mai Hospital, Hanoi 100000, Vietnam; phamthethach@gmail.com; 6 Center for Tropical Diseases, Bach Mai Hospital, Hanoi 100000, Vietnam; thai2vn@gmail.com (N.Q.T.); anngovth@gmail.com (N.V.A.); 7 Infection Control Department, Bach Mai Hospital, Hanoi 100000, Vietnam; thuksnk@gmail.com; 8 Institute for Preventive Medicine and Public Health, Hanoi Medical University, Hanoi 100000, Vietnam; donamkhanh@hmu.edu.vn; 9 General Hospital of Quang Nam Province, Quang Nam 560000, Vietnam; bvdkkvquangnam@gmail.com; 10 General Hospital in the Northern Mountainous Region of Quang Nam, Quang Nam 560000, Vietnam; thstomuoi@gmail.com; 11 Hoa Vang General Hospital, Da Nang 550000, Vietnam; Daivinh75@gmail.com; 12 Da Nang C Hospital, Da Nang 550000, Vietnam; nnguyentrongthien@gmail.com (N.T.T.); drnhandanang@gmail.com (L.D.N.)

**Keywords:** healthcare workers, mental health, psychological, COVID-19, Vietnam

## Abstract

Adopting a cross-sectional study design, we aimed to examine the prevalence of psychological problems in different healthcare workers during the COVID-19 pandemic in the hospitals in these COVID-19 hotspots (Da Nang city and Quang Nam province) and to explore the socioeconomic and COVID-19 control-related factors that are associated with various psychological problems. A total of 611 healthcare workers were included in the final analysis from 1 August 2020 to 31 August 2020. The prevalence of anxiety, depression, insomnia, and overall psychological problems was 26.84%, 34.70%, 34.53%, and 46.48%, respectively. The prevalence rates of anxiety were approximately equal amongst the groups of healthcare workers, and moderate-to-severe anxiety was the most common in physicians (11.11%). The prevalence of depression was the highest in nurses (38.65%) and moderate-to-severe depression was mainly found in physicians (11.81%). The prevalence rates of insomnia were 34.03% in physicians, 36.20% in nurses, and 31.21% in technicians; in particular, the rate of moderate-to-severe insomnia was higher in physicians and nurses compared to technicians. The prevalence of overall moderate-to-severe psychological problems was the highest among physicians (14.58%), followed by nurses (12.58%) and technicians (9.22%). Statistically significant associated factors of current psychological problems were the occupations of physicians or nurses, less than 1 year of experience, university education, living with 4–5 people, reporting 1000–5000 m distance between home and workplace, participating in the COVID-19 control for less than 1 week, being under social isolation at home, being affected a lot by the community, reporting inadequate equipment in current workplace conditions, frequently working in the department directly in contact with the COVID-19 patients, and feeling anxious, stressed, or sad about current works. Present findings can provide valuable evidence for the policymakers and managers to adopt supportive, encouraging, motivational, protective, training, and educational interventions into healthcare workforce in other parts of Vietnam.

## 1. Introduction

A major virus outbreak caused by the novel coronavirus (SARS-CoV-2) in the 21st century, known as coronavirus 2019 (COVID-19) pandemic, has created a tremendous and unprecedented public health crisis globally [1,2]. Despite comparatively low COVID-19 mortality rate, a number of reported deaths due to COVID-19 around the world has been dramatically higher than those by the previous severe acute respiratory syndrome (SARS) and Middle East respiratory syndrome combined [3]. With larger number of new COVID-19 cases and deaths per day, this global COVID-19 pandemic poses great emergency situations to the local medical officials as well as their healthcare systems. Obviously, medical resources and medical care services also increase by unprecedented demands and are easily placed at maximum capacity. With limited resources in extremely long working hours in high-pressure environments, front-line healthcare workers have a much higher risk of getting mental health issues [4,5]. Vietnam has been extensively recognized across the world successes in COVID-19 control. This country’s early successful experience in COVID-19 response provides useful lessons particularly for low-income and middle-income countries. Herein, Vietnam, following the implementation of early lockdown strategy, was one of the few countries achieving effective control of COVID-19 outbreak [6]. A “stay-at-home” order was, under Directive No. 16/CT-TTg issued on 31 March 2020, imposed nationwide to curb the spread of the contagion and alleviate the pressure on the entire healthcare system. The effectiveness in outbreak control was evidenced by no community transmission within 99 days since April 15 [7]. Therefore, while strict COVID-19 lockdown orders in many countries had still been in effect, the Government of Vietnam relaxed social distancing rules for almost all provinces and cities on 23 April [8]. The early success in Vietnam’s epidemic control has mainly been attributed to the national emergency response across the whole sociopolitical system; in particular, a 22-day nationwide lockdown. The duration with no COVID-19 community transmission ended on 25 July when cases were reported in Da Nang City—the biggest tourist city in the country with >1 million local citizens and ~8 million tourists annually [9]. This disruption made the COVID-19 pandemic in Vietnam more unforeseeable than ever [10,11,12]. Immediately, contact tracing efforts, epidemiologic investigations, and other public health responses were strengthened under the strong guidance of the Vietnamese Prime Minister Nguyen Xuan Phuc, yet the source of the infection is not known in several new cases [12,13].

In such unexpected situations, no study was conducted in Vietnam’s second wave of COVID-19 to investigate the distribution of psychological problems among different groups of healthcare workers (the second wave of COVID-19 started in Vietnam on 25 July, which was mainly concentrated in two COVID-19 hotspots including Da Nang city and Quang Nam province). Importantly, compared with the general population, healthcare workers have been facing tremendous pressure from COVID-19 with significant shortages of staff and medical supplies [14,15,16], which increased the incidence of psychological problems such as fear, anxiety, depression, and insomnia among healthcare workers, and further can cause the decrease in long-term quality of life [17,18]. In the previous SARS epidemic, a high degree of emotional distress was documented in 29–35% of hospital workers [16]. Several years later, up to 10% of them still had self-reported symptoms of post-traumatic stress [19]. Hence, there is increasing interest in the prevalence and impact of psychological problems on healthcare workers with the aim of promptly dealing with mental health issues and providing psychological support to healthcare workers.

Given the insights acquired from the previous global outbreaks and their psychosocial impacts according to the context of phased COVID-19 control in each country, an early assessment of medical staff’s mental health in Vietnam was vital to consider appropriate psychological interventions in the resource-scarce conditions. Furthermore, a more comprehensive understanding of psychological burden amongst different groups of healthcare workers in COVID-19 hotspots in the second wave of Vietnam needs to be prioritized for providing specific psychological support, improving mental health support services and strengthening mental healthcare in subsequent outbreaks [20]. In this context, this cross-sectional study was promptly conducted to investigate the prevalence of psychological problems in different healthcare workers during the COVID-19 pandemic in the hospitals in these COVID-19 hotspots (Da Nang city and Quang Nam province) and to explore potential factors associated with various psychological problems.

## 2. Materials and Methods

### 2.1. Study Design and Sampling Methods

We carried out an online cross-sectional study on healthcare workforce between 1 August 2020 and 31 August 2020. Participants who met the following criteria were included in the study: (1) age from 18 and over; (2) being healthcare professionals including physicians, nurses, and technicians; (3) working at one of the Hospitals in Da Nang city and Quang Nam province; and (4) agreeing to participate in the survey by providing an online informed consent.

No material incentives were suggested to the respondents for their engagement of the survey to avoid them from answering more than once. In total, the responses of 611 healthcare workers were included in the final analysis from 1 August 2020 to 31 August 2020.

We designed a structured questionnaire via Google form. We applied the “snowball sampling technique” to recruit participants. Several core groups of the board of directors of hospitals (also known as physicians) from a hospital in Da Nang city and a hospital in Quang Nam province have been centered upon at the beginning of the recruitment process. The core groups sent the link to colleagues at hospitals in Da Nang city and Quang Nam province to access the questionnaire. The individuals who had been involved in the study were instructed to invite other colleagues to join because they were more likely to know other people who have similar background characteristics and are suitable to be involved in the survey. One person could easily send the link of survey via weblink, email, social network, and messenger apps to others.

### 2.2. Measurements

A questionnaire was developed by a group of psychiatrists at the National Institute of Mental Health (Hanoi, Vietnam) to collect data on socioeconomic characteristics and psychological status of healthcare workers and information about the COVID-19 pandemic. Healthcare workers’ psychological problems were assessed with the use of the Vietnamese versions of the Insomnia Severity Index (ISI) scale, the 9-item Patient Health Questionnaire (PHQ-9), and the 7-item Generalized Anxiety Disorder-7 (GAD-7) scale.

#### 2.2.1. PHQ-9

The PHQ-9 scale is a 9-item depression questionnaire that is designed to detect probable individuals of depression and to assess degree of depression severity within past two weeks. In various medical settings, the validated depression scale was reported with good reliability (Cronbach’s α = 0.86–0.89). The total score of PHQ-9 scale after self-reported response ranges from 0 to 27, and more severe depression symptom was shown with a higher score. The scores were categorized as follows: absence of depression (0–4), mild depression (5–9), moderate depression (10–14), and severe depression (15–27).

#### 2.2.2. GAD-7

The GAD-7 scale, a 7-item self-reported anxiety questionnaire, proved valid with high reliability (Cronbach’s α = 0.89). Though initially designed to identify generalized anxiety disorder (GAD), the GAD-7 has also been considered as a good screening tool for other common anxiety disorders. All the items are rated on a 4-point scale, and the total score ranges from 0 to 21 and is interpreted as follows: absence of anxiety (0–4), mild anxiety (5–9), moderate anxiety (10–14), and severe anxiety (15–21).

#### 2.2.3. ISI

The ISI is a valid self-reported instrument with the high reliability of the scale (Cronbach’s α = 0.90–0.91). This scale was used to diagnose insomnia and to categorize the level of symptom severity in the previous 2 weeks. A 5-point Likert scale is used to rate the 7 items. The total score of ISI scale ranges from 0 to 28, more severe insomnia was reported with a higher score, and the total score is categorized as follows: absence of insomnia (0–7), mild insomnia (8–14), moderate insomnia (15–21), and severe insomnia (22–28).

### 2.3. Variables

#### 2.3.1. Main Outcome Variables

The main study outcome variable (or dependent variable) was whether a person had any psychological problems. There were four dependent variables, in detail, defined as follows:

○ *Anxiety* was defined as that in which an individual had a GAD-7 score of ≥10 [21].
○ *Depression* was defined as that in which an individual had a PHQ-9 score of ≥10 [22].
○ *Clinical insomnia* was defined as that in which an individual had an ISI score of ≥15 [23].
○ *Overall psychological problem* was defined as that in which an individual had any symptom of moderate/severe anxiety, depression, or insomnia.

#### 2.3.2. Socioeconomic and COVID-19 Control-Related Variables

The participants self-reported socioeconomic data including gender (male/female), age (years), occupation (physician/nurse/technician), education (lower secondary/upper secondary/college/university/postgraduation), marital status (married/single/widowed/divorced), living area (village/town/city), professional experience (years), number of people living with (people), distance between home and main or regular place of work (meter).

In addition, several main COVID-19 control-related data that were also responded to by healthcare workers included current situation (being in quarantine zone doing nothing/being in quarantine to care for the COVID-19 infected patient/being in quarantine at home/having ended the duration in quarantine and normally work/normal working), duration participating in COVID-19 control (weeks), working directly in contact with COVID-19 patients frequently (no/yes), level of preparation before participating in COVID-19 control (none/average/adequate), level of equipment in current workplace conditions (none/average/adequate), affected by workplace conditions (no/yes), affected a lot by the community (no/yes), feeling stressed about current works (no/yes), feeling anxious about current work (no/yes), feeling sad about current works (no/yes).

### 2.4. Data Analysis

We used descriptive statistical analysis to characterize the samples of healthcare workers. Frequencies and proportions for each categorical variable were calculated and described, while only age variable was expressed as mean and standard deviation (SD). According to occupation (physician/nurse/technician), the prevalence of symptoms of anxiety, depression, insomnia, and the overall psychological problems was reported with their categories. For the four dependent variables that included anxiety, depression, insomnia, and overall psychological problem as defined above, we firstly applied the univariate analysis using logistic regression to identify factors associated with four outcomes. Then, the multivariate logistic regression was utilized to identify independent associated factors for the four above outcome variables, followed by stepwise backward selection strategies that used a log-likelihood ratio test at a *p*-value of 0.2 to obtain reduced models. Hence, a total of four reduced models were reported. Odds ratios with 95% confidence intervals were constructed. *p* < 0.05 was considered statistically significant. Stata 13.1 software (StataCorp LLC, USA) was used for statistical analyses.

## 3. Results

### 3.1. Socioeconomic Characteristics and COVID-19 Control-Related Characteristics in Healthcare Workers

Data from a total of 611 eligible participants were included in the final analysis, for a participation rate of 87.16% (611 of 701 participants). Table 1 presents the socioeconomic and COVID-19 control-related characteristics of the participants. At the time of current survey, half of the participants had been still working normally and had never been in the quarantine zone (50.25%). Most healthcare workers participated in COVID-19 control within 1–4 week(s). Approximately three-fourths of the participants were female (*n* = 453, 74.14%). The mean age of participants was 32.49 ± 8.35 years old. Most of the participants reported the education of college or higher (96.89%) and over 3 years of profesional experience (83.63%). Of participants, 71.52% were married at the time of the study, while 27.33% were single. A total of 77.91% participants were living with four people or more. More than 80% of participants reported that the distance from home to main or regular place of work was more than 1000 m. 

### 3.2. Prevalence of Psychological Problems in Healthcare Workers

**Figure 1:**
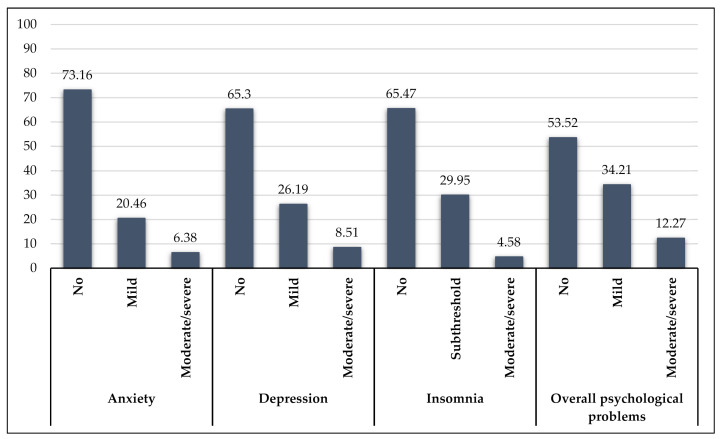
Severity categories of depression, anxiety, and insomnia and overall psychological problems.

The prevalence of symptoms for the three mental health conditions among the total sample was 26.84% for anxiety (including 20.46% with mild anxiety and 6.38% with moderate-to-severe anxiety), 34.70% for depression (including 26.19% with mild depression and 8.51% with moderate-to-severe depression), and 34.53% for insomnia (including 29.95% with subthreshold insomnia and 4.58% with moderate-to-severe insomnia). The prevalence of overall psychological problems was 46.48% (including 34.21% with mild level and 12.27% with moderate-to-severe level) (Figure 1). As was shown in Table 2, the prevalence rates of anxiety were approximately equal amongst the groups of healthcare workers, and moderate-to-severe anxiety was the most common in physicians (11.11%). The prevalence of depression was the highest in nurses (38.65%) and moderate-to-severe depression was mainly found in physicians (11.81%). The prevalence rates of insomnia were 34.03% in physicians, 36.20% in nurses, and 31.21% in technicians, in particular, the rate of moderate-to-severe insomnia was higher in physicians and nurses compared to technicians. The prevalence of overall moderate-to-severe psychological problems was the highest among physicians (14.58%), followed by nurses (12.58%), and technicians (9.22%).

### 3.3. Factors Associated with Psychological Problems

The results of univariate analysis of socioeconomic and epidemic-related variables are presented in Table 3. Married status, 5000–10,000 m distance between the home and workplace, adequate level of preparation before participating in COVID-19 control, being affected by workplace condition, being affected a lot by the community, feeling stressed about current work, feeling anxious about current work, and feeling sad about current work were significantly associated with self-reported anxiety. Variables showing significant association with self-reported depression included having over 10 years of professional experience, an adequate level of equipment in current workplace conditions, being affected a lot by the community, feeling stressed about current work, feeling anxious about current work, and feeling sad about current work. Significant associations of self-reported insomnia were age, working in the department directly in contact with COVID-19 patients frequently, level of preparation before participating in COVID-19 control, being affected by workplace conditions, being affected a lot by the community, feeling anxious about current work, and feeling sad about current work. In addition, variables that were associated with overall psychological problems included living area, working in the department directly in contact with COVID-19 patients frequently, level of preparation before participating in COVID-19 control, level of equipment in current workplace conditions, being affected by workplace conditions, being affected a lot by the community, feeling stressed about current work, feeling anxious about current work, and feeling sad about current work.

Four multivariate logistic regression models were shown in Table 4. Independently associated factors that affected the prevalence of anxiety included type of health workers and being affected a lot by the community. Nurses and technicians had significantly lower odds of having anxiety compared to physicians (OR 0.40; 95%CI: 0.18–0.90 and OR 0.08; 95%CI: 0.02–0.44 respectively). Being affected a lot by the community was associated with a higher risk of having anxiety (OR 5.39; 95%CI: 1.70–17.11).

In depression model (Table 4), individuals working as nurses had lower likelihood of having depression than those of physicians (OR 0.38; 95%CI: 0.18–0.80). Respondents who reported professional experience of over 10 years had significantly lower odds of having depression compared to those with less than 1 year of experience (OR 0.24; 95%CI: 0.07–0.84). Participants reporting average and a good level of equipment in current workplace conditions were significantly associated with a lower risk of having depression compared to those reporting a poor level of equipment (OR 0.19; 95%CI: 0.05–0.69 and OR 0.12; 95%CI: 0.03–0.47 respectively). Beside this, living with 4–5 people (OR 2.11; 95%CI: 1.07–4.14), feeling anxious about current works (OR 2.52; 95%CI: 1.07–5.92), and feeling sad about current works (OR 3.71; 95%CI: 1.72–7.98) were demonstrated as elevated risk factors for depression.

Individuals being nurses (OR 3.69; 95%CI: 1.14–11.93) and 1000–5000 m distance between home and workplace (OR 2.77; 95%CI: 1.05–7.29) were associated with a higher risk of having self-reported insomnia. Compared to respondents reporting education of lower secondary/upper secondary, health workers with university education had an elevated risk for insomnia (OR 7.88; 95%CI: 1.37–45.30). Those working in the department directly in contact with COVID-19 patients frequently scored 3.39 (95%CI: 1.17–9.86) times higher for insomnia. However, individuals who participated in COVID-19 control for 2–4 weeks were as less risk of insomnia than those participating for less than 1 week (OR 0.28; 95%CI: 0.09–0.89).

In reduced multivariate logistic model, elevated risk factors for overall psychological problems included 1000–5000 m distance between home and workplace (OR 1.93; 95%CI: 1.08–3.45), being in quarantine at home (OR 2.28; 95%CI: 1.01–5.16), feeling stressed about current works (OR 4.84; 95%CI: 2.40–9.75), and feeling sad about current works (OR 3.24; 95%CI: 1.76–6.00). However, those living in city had the lower risk of overall psychological problems than those in village (OR 0.45; 95%CI: 0.24–0.86) (Table 4).

## 4. Discussion

From a large-sample survey in Da Nang city and Quang Nam province in the second wave of COVID-19, we investigated the prevalence of anxiety, depression, insomnia, and the overall psychological problems in healthcare workers during the COVID-19 pandemic. In these COVID-19 hotspots, the results were 26.84%, 34.70%, 34.53%, and 46.48%, respectively. Using the same scales in measurement of mental health symptoms in the healthcare workforce population, Jianyu Que’s study indicated more common symptoms of anxiety (46.04%), depression (44.37%), and overall psychological problems (56.59%) (excluding insomnia) [24]. However, with extensive geographic coverage across China, Le Shi [25] reported significantly higher prevalence of these mental health symptoms in the general population compared to Jianyu Que’s and our research [24]. Our prevalence of the symptoms of mental health was less common compared to China’s reports, consistent with the complexity level of the epidemic situation in each country. Of note, the main epidemiological difference is that, while COVID-19 outbreaks occur in almost all provinces of China [26], COVID-19 outbreaks only emerge in several tourism hospots such as Da Nang city, Quang Nam province, Hanoi city and Ho Chi Minh city [27]. The COVID-19 pandemic caused physicians to look more closely at many aspects of their profession under available work pressure. However, possibly because of an earlier proven system of epidemic control in Vietnam, there are no discernible disparities in overall moderate-to-severe psychological problems being reported, with 14.58% in physicians, 12.58% in nurses and 9.22% in technicians. We found that prevalence rates of anxiety, depression, and insomnia variously changed amongst different kinds of health workers. The prevalence rates of anxiety were approximately equal amongst the groups of healthcare workers, while nurses had the most common symptoms of both depression (38.65%) and insomnia (36.02%). This result is consistent with the characteristics of the nursing profession. The nurses are mostly women who are more susceptible to higher workloads, with more healthcare needs and greater risk of direct exposure to patients with COVID-19 [28]. In keeping with Vietnamese woman’s culture, these nurses also care for their families [26]. To date, no studies have comprehensively investigated the prevalence of their mental health during a COVID-19 pandemic in Vietnam. Nevertheless, a nationwide population-based research that follows the present study is still required to provide more solid evidence from resource-varied condition.

In response to increasing healthcare needed to deal with COVID-19 worldwide, the known reasons for the psychological distress to which medical health workers were exposed might be related to the many difficulties of being safe at work, such as the initially insufficient understanding of the virus, the lack of prevention and control knowledge, the long-term workload, the high risk of exposure to patients with COVID-19, the shortage of medical protective equipment [29], the lack of rest, and the exposure to critical life events [30] such as death. Herein, we identified a variety of the risk factors associated with different psychological problems. Our study revealed the individuals working as nurses had lower likelihood of having symptoms of anxiety and depression than those of physicians; however, nurses were associated with a higher risk of having self-reported insomnia. This can be partially explained by the constant daily impact of workload on burnout and performance as well as typically the work of continuous care for the patient. Though anxious and unsafe feelings are an instinctive reaction to the changes of environment, there may be a significant difference when dissecting individual population groups. It is difficult for us to give a plausible explanation for the increase in the symptoms of anxiety and depression among the physician group, but one prompt assessment of the current extent of insomnia is comprehensive to obtain the most general picture of psychological distress in healthcare workforce. In several recent reports under the COVID-19 pandemic mid and early period, the psychosocial problems were only evaluated in the whole group of health workers but did not compare these prevalences between medical officer patterns like our study [31], while others indicated nurses experienced more severe symptom levels of depression and anxiety than physicians [32,33]. The reasons for this might be due to the differences amongst countries in managing their workload, providing emotional support and responding to their personal needs, impacting the health worker’s levels of fear, social isolation and work stress. Towards self-reported insomnia, similar findings were found in Nepal [32] and China [33]. Current evidence suggested a need for psychologically-specific interventions according to mental health disorder categories among the types of health workers, especially for those working as physicians in these hotspots. Of note, we found that respondents with overall psychological problems were more likely to report feeling stressed and sad about current works, suggesting that health authorities at the unit, hospital, and city levels should pay more attention to those populations. 

Although successful use of quarantine has been proved as a public health measure in Vietnam, quarantine is often an unpleasant experience because of separation from loved ones, the loss of freedom, uncertainty over disease status, and boredom [34,35]. Our multivariate regression model also indicated the evidence of a significant association between overall psychological problems and the *social isolation at home*. This result was consistent when recent literature that indicated periods of isolation can have long-term effects, with the presence—up to 3 years later—of psychiatric symptoms [36]. Further, prolonged isolation can adversely affect physical and emotional health, altering sleep and nutritional rhythms, as well as reducing opportunities for movement; nevertheless, quarantine duration was not analyzed in this paper. Hence, further studies in which clear and precise information is explored in detail on psychological consequences of social isolation during COVID-19 pandemic are required.

As is mentioned in the literature, the environment can play a vital role in maintaining healthy emotions and sleep [37,38]. The present study clearly revealed individuals who reported frequently working in the department directly in contact with COVID-19 patients were likely to develop the symptoms of insomnia. The isolation of these individuals, themselves a high-risk group for COVID-19 infection, in separate care areas for COVID-19 patients, potentially contributes to poor mental health consequences. Facilities should establish a better attention level of care for potential SARS-CoV-2 spread in this group within the facility, following specific recommendations of mental health, thereby, maintaining a low threshold for the symptoms of psychological disorders in staff with high-risk exposures.

Although this study did not report an extensive geographic coverage across Vietnam and a large sample size, to the best of our knowledge, this is the first study that has systematically investigated the prevalence of, and factors associated with, mental health symptoms (i.e., symptoms of depression, anxiety, insomnia, and overall psychological problems) by standardized rating scales of National Institute of Mental Health for the Vietnamese general population during the COVID-19 pandemic. Importantly, several limitations need be acknowledged in this study. This was a cross-sectional online survey and the sample is not necessarily a best representation. The causal relationships should be interpreted with caution. Using the snowball sampling method might lessen the representability of our study. In return, our findings are urgent and consistent with the urgent requirements of the epidemic situation in Vietnam. The self-reported data collection might lead to recall bias. More studies are needed to explore the longitudinal trajectories of anxiety, depression, and insomnia symptoms in healthcare workers during the COVID-19 pandemic in Vietnam. The number of physicians, nurses, and technicians who participated in this survey was limited, which may limit the generalizability of findings. The results were based on self-reported questionnaires that investigated psychological problems, which might lead to recall bias and be different from clinical diagnostic interviews. Finally, this study is not capable of accounting any pre-existing medical condition and analysing possible association with socio-economic burden.

## 5. Conclusions

The initital report in two Vietnam’s COVID-19 hotspots (Da Nang and Quang Nam) showed the relatively high prevalence of anxiety, depression, insomnia and the overall psychological problems in healthcare workforce, especially among the groups of physicians or nurses, people with less than 1 year experience, having university education, living with 4–5 people, reporting 1000–5000 m distance between home and workplace, participating in COVID-19 control for less than 1 week, being under *social isolation at home*, being affected a lot by the community, those reporting inadequate equipment in current workplace conditions, frequently working in the department directly in contact with COVID-19 patients, and feeling anxious or stressed or sad about current works. During the past outbreaks, thousands of healthcare workers had to fight with this disease in the front-line. Lessons learned from this current analysis can provide valuable findings for the policymakers and managers to adopt supportive, encouraging, motivational, protective, training, and educational interventions in the healthcare workforce in other parts of Vietnam.

## Figures and Tables

**Table 1:** Socioeconomic and COVID-19 control-related characteristics of the respondents.

Characteristics	Physician (*n* = 144)	Nurses (*n* = 326)	Technicians (*n* = 141)	Total (*n* = 611)
	Count (% of Total)	Count (% of Total)	Count (% of Total)	Count (% of Total)
Gender				
Male	83 (57.64)	23 (7.06)	52 (36.88)	158 (25.86)
Female	61 (42.36)	303 (92.94)	89 (63.12)	453 (74.14)
Marital status				
Married	93 (64.58)	245 (75.15)	99 (70.21)	437 (71.52)
Single	50 (34.72)	78 (23.93)	39 (27.66)	167 (27.33)
Widowed	0 (0.00)	1 (0.31)	1 (0.71)	2 (0.33)
Divorced	1 (0.69)	2 (0.61)	2 (1.42)	5 (0.82)
Number of people living with (people)				
1–3 people	37 (25.69)	66 (20.25)	32 (22.70)	135 (22.09)
4–5 people	74 (51.39)	166 (50.92)	73 (51.77)	313 (51.23)
>5 people	33 (22.92)	94 (28.83)	36 (25.53)	163 (26.68)
Living area				
Village	41 (28.47)	143 (43.87)	58 (41.13)	242 (39.61)
Town	39 (27.08)	58 (17.79)	42 (29.79)	139 (22.75)
City	64 (44.44)	125 (38.34)	41 (29.08)	230 (37.64)
Distance between home and main or regular place of work (meter)				
<1000 m	32 (22.22)	57 (17.48)	27 (19.15)	116 (18.99)
1000–5000 m	43 (29.86)	102 (31.29)	52 (36.88)	197 (32.24)
5000–10,000 m	32 (22.22)	64 (19.63)	31 (21.99)	127 (20.79)
5000–10,000 m	37 (25.69)	103 (31.60)	31 (21.99)	171 (27.99)
Education				
Lower secondary/upper secondary	0 (0.00)	18 (5.52)	1 (0.71)	19 (3.11)
College	2 (1.39)	181 (55.52)	90 (63.83)	273 (44.68)
University	79 (54.86)	123 (37.73)	43 (30.50)	245 (40.10)
Postgraduation	63 (43.75)	4 (1.23)	7 (4.96)	74 (12.11)
Professional experience (years)				
<1 year	6 (4.17)	9 (2.76)	6 (4.26)	21 (3.44)
1–2 year(s)	25 (17.36)	37 (11.35)	17 (12.06)	79 (12.93)
3–5 years	37 (25.69)	65 (19.94)	36 (25.53)	138 (22.59)
5–10 years	35 (24.31)	112 (34.36)	45 (31.91)	192 (31.42)
>10 years	41 (28.47)	103 (31.60)	37 (26.24)	181 (29.62)
Current situation				
Being in quarantine zone doing nothing	26 (18.06)	32 (9.82)	9 (6.38)	67 (10.97)
Being in quarantine to care for the COVID-19 infected patient	28 (19.44)	55 (16.87)	8 (5.67)	91 (14.89)
Being in quarantine at home	29 (20.14)	33 (10.12)	7 (4.96)	69 (11.29)
Having ended the duration in quarantine and normally work	17 (11.81)	36 (11.04)	24 (17.02)	77 (12.60)
Normal working	44 (30.56)	170 (52.15)	93 (65.96)	307 (50.25)
Duration participating in COVID-19 control (weeks)				
<1 week	30 (20.83)	53 (16.26)	27 (19.15)	110 (18.00)
1–2 week(s)	45 (31.25)	127 (38.96)	50 (35.46)	222 (36.33)
2–4 weeks	54 (37.50)	112 (34.36)	43 (30.50)	209 (34.21)
4–8 weeks	6 (4.17)	15 (4.60)	11 (7.80)	32 (5.24)
>8 weeks	9 (6.25)	19 (5.83)	10 (7.09)	38 (6.22)
	**Mean (SD)**	**Mean (SD)**	**Mean (SD)**	**Mean (SD)**
Age (years)	34.49 (9.63)	31.63 (7.45)	32.41 (8.63)	32.49 (8.35)

SD: standard deviation.

**Table 2:** Prevalence of psychological problems according to the groups of healthcare workers.

	Anxiety			Depression			Insomnia			Overall Psychological Problems		
	No *n* (%)	Mild *n* (%)	Moderate/ Severe *n* (%)	No, *n* (%)	Mild *n* (%)	Moderate/ Severe *n* (%)	No *n* (%)	Subthreshold *n* (%)	Moderate/ Severe *n* (%)	No *n* (%)	Mild *n* (%)	Moderate/ Severe *n* (%)
Physician, *n* (% of total)	107 (74.31)	21 (14.58)	16 (11.11)	97 (67.36)	30 (20.83)	17 (11.81)	95 (65.97)	42 (29.17)	7 (4.86)	78 (54.17)	45 (31.25)	21 (14.58)
Nurse, *n* (% of total)	235 (72.09)	70 (21.47)	21 (6.44)	200 (61.35)	103 (31.60)	23 (7.06)	208 (63.80)	99 (30.37)	19 (5.82)	164 (50.31)	121 (37.12)	41 (12.58)
Technician, *n* (% of total)	105 (74.47)	34 (24.11)	2 (1.42)	102 (72.34)	27 (19.15)	12 (8.51)	97 (68.79)	42 (29.79)	2 (1.42)	85 (60.28)	43 (30.50)	13 (9.22)

**Table 3:** Univariable logistic regression analysis of the factors associated with psychological problems.

Variable	Anxiety	Depression	Insomnia	Overall Psychological Problems
	OR (95% CI)	OR (95% CI)	OR (95% CI)	OR (95% CI)
Age	0.99 (0.95–1.03)	0.98 (0.95–1.02)	0.93 (0.86–0.99) *	0.98 (0.95–1.01)
Gender				
Male	REF	REF	REF	REF
Female	1.01 (0.48–2.13)	1.05 (0.55–2.02)	1.64 (0.61–4.38)	1.46 (0.80–2.64)
Marital status				
Single	REF	REF	REF	REF
Married	2.65 (1.02–6.90) *	1.04 (0.55–1.97)	0.80 (0.35–1.80)	1.32 (0.75–2.35)
Widowed	-	-	-	-
Divorced	8.1 (0.76–86.23)	-	-	2.21 (0.23–20.89)
Number of people living with (people)				
1–3 people	REF	REF	REF	REF
4–5 people	1.18 (0.48–2.88)	2.01 (0.86–4.69)	1.65 (0.54–5.06)	1.88 (0.91–3.88)
>5 people	1.58 (0.61–4.09)	1.72 (0.67–4.39)	1.91 (0.58–6.36)	2.16 (0.99–4.69)
Living area				
Village	REF	REF	REF	REF
Town	1.18 (0.55–2.52)	0.97 (0.49–1.94)	1.22 (0.51–2.93)	0.93 (0.52–1.68)
City	0.51 (0.22–1.15)	0.52 (0.26–1.04)	0.47 (0.18–1.26)	0.47 (0.26–0.85) *
Distance between home and main or regular place of work (meter)				
<1000 m	REF	REF	REF	REF
1000–5000 m	1.07 (0.47–2.39)	1.14 (0.53–2.46)	0.88 (0.35–2.21)	1.26 (0.63–2.49)
5000–10,000 m	0.17 (0.04–0.79) *	0.64 (0.25–1.66)	0.22 (0.04–1.04)	0.62 (0.27–1.46)
5000–10,000 m	0.59 (0.23–1.50)	0.72 (0.31–1.69)	0.49 (0.17–1.45)	1.08 (0.53–2.20)
Education				
Lower Secondary/Upper Secondary	REF	REF	REF	REF
College	1.35 (0.17–10.64)	1.73 (0.22–13.57)	-	1.29 (0.29–5.82)
University	0.93 (0.11–7.54)	1.51 (0.19–11.96)	-	1.09 (0.24–5.00)
Postgraduation	1.88 (0.22–16.29)	2.18 (0.26–18.60)	-	1.18 (0.23–5.96)
Professional experience (years)				
<1 year	REF	REF	REF	REF
1–2 year(s)	0.38 (0.06–2.41)	0.48 (0.13–1.78)	-	0.69 (0.19–2.43)
3–5 years	0.74 (0.15–3.65)	0.30 (0.08–1.07)	-	0.48 (0.14–1.63)
5–10 years	0.75 (0.16–3.54)	0.55 (0.17–1.78)	-	0.76 (0.24–2.41)
>10 years	0.56 (0.11–2.73)	0.22 (0.06–0.80) *	-	0.44 (0.13–1.46)
Current situation				
Being in quarantine zone doing nothing	REF	REF	REF	REF
Being in quarantine to care for the COVID-19 infected patient	1.36 (0.43–4.26)	1.03 (0.31–3.41)	3.57 (0.74–17.08)	2.01 (0.73–5.48)
Being in quarantine at home	1.40 (0.42–4.65)	1.63 (0.50–5.25)	2.00 (0.35–11.30)	1.93 (0.67–5.55)
Having ended the duration in quarantine and normally work	0.68 (0.17–2.64)	1.85 (0.60–5.72)	2.75 (0.54–14.09)	2.26 (0.82–6.26)
Normal working	0.59 (0.21–1.71)	0.96 (0.35–2.63)	0.76 (0.15–3.73)	1.06 (0.42–2.67)
Duration participating in COVID-19 control (weeks)				
<1 week	REF	REF	REF	REF
1–2 week(s)	0.99 (0.41–2.39)	1.16 (0.53–2.52)	1.07 (0.42–2.70)	1.15 (0.59–2.27)
2–4 weeks	0.85 (0.34–2.11)	0.89 (0.39–2.01)	0.36 (0.11–1.16)	0.89 (0.44–1.80)
4–8 weeks	0.85 (0.17–4.22)	0.32 (0.04–2.62)	-	0.71 (0.19–2.64)
>8 weeks	-	0.27 (0.03–2.18)	0.40 (0.05–3.34)	0.38 (0.08–1.76)
Working directly in contact with COVID-19 patients frequently				
No	REF	REF	REF	REF
Yes	1.85 (0.89–3.85)	1.57 (0.81–3.05)	2.65 (1.19–5.91) *	1.93 (1.10–3.35) *
Level of preparation before participating in COVID-19 control				
None	REF	REF	REF	REF
Average	0.57 (0.21–1.59)	0.76 (0.28–2.09)	0.78 (0.22–2.73)	0.79 (0.33–1.89)
Adequate	0.20 (0.06–0.67) **	0.35 (0.12–1.07)	0.20 (0.04–0.94) *	0.34 (0.13–0.89) *
Level of equipment in current workplace conditions				
None	REF	REF	REF	REF
Average	0.73 (0.16–3.33)	0.23 (0.08–0.64) **	1.18 (0.15–9.29)	0.35 (0.12–0.97) *
Adequate	0.31 (0.06–1.53)	0.10 (0.03–0.31) ***	0.39 (0.04–3.41)	0.17 (0.06–0.50) **
Affected by workplace conditions				
No	REF	REF	REF	REF
Yes	4.18 (1.72–10.13) **	1.83 (0.98–3.41)	6.26 (1.87–20.95) **	2.28 (1.32–3.95) **
Affected a lot by the community				
No	REF	REF	REF	REF
Yes	11.60 (4.07–33.06) ***	2.62 (1.43–4.79) **	16.72 (3.93–71.11) ***	3.50 (2.06–5.96) ***
Feeling stressed about current works				
No	REF	REF	REF	REF
Yes	6.83 (3.08–15.14) ***	6.85 (3.44–13.62) ***	-	8.84 (4.81–16.23) ***
Feeling anxious about current works				
No	REF	REF	REF	REF
Yes	4.98 (2.47–10.06) ***	6.37 (3.36–12.07) ***	13.33 (4.56–38.96) ***	5.66 (3.35–9.57) ***
Feeling sad about current works				
No	REF	REF	REF	REF
Yes	5.62 (2.89–10.96) ***	7.62 (4.18–13.89) ***	10.87 (4.67–25.33) ***	7.03 (4.21–11.74) ***

OR: odds ratio; CI: confidence interval; REF: reference *, **, ***: significant at 0.05, 0.01, and 0.001.

**Table 4:** Multivariable logistic regression analysis of the factors associated with psychological problems.

Variable	Anxiety	Depression	Insomnia	Overall Psychological Problems
	OR (95% CI)	OR (95% CI)	OR (95% CI)	OR (95% CI)
Age		1.06 (0.98–1.13)	0.93 (0.85–1.02)	
Gender				
Male		REF		REF
Female		1.89 (0.79–4.47)		
Heathcare workforce				
Physician	REF	REF	REF	
Nurse	0.40 (0.18–0.90) *	0.38 (0.18–0.80) *	3.69 (1.14–11.93) *	
Technician	0.08 (0.02–0.44) **			
Marital status				
Single	REF			REF
Married	2.76 (0.95–8.03)			
Widowed				
Divorced	13.82 (0.97–196.30)			
Number of people living with (people)				
1–3 people		REF		REF
4–5 people		2.11 (1.07–4.14) *		1.45 (0.83–2.54)
>5 people				
Living area				
Village	REF			REF
Town				
City	0.46 (0.19–1.10)			0.45 (0.24–0.86) *
Distance between home and main or regular place of work (meter)				
<1000 m	REF	REF	REF	REF
1000–5000 m	2.16 (0.99–4.71)	1.95 (0.99–3.85)	2.77 (1.05–7.29) *	1.93 (1.08–3.45) *
5000–10,000 m	0.33 (0.07–1.54)			
5000–10,000 m				
Education				
Lower Secondary/Upper Secondary	REF		REF	REF
College	0.58 (0.26–1.32)			
University			7.88 (1.37–45.30) *	
Postgraduation				
Professional experience (years)				
<1 year		REF	REF	REF
1–2 year(s)			3.69 (0.96–14.24)	
3–5 years		0.47 (0.20–1.13)		0.54 (0.27–1.08)
5–10 years				
>10 years		0.24 (0.07–0.84) *		
Current situation				
Being in quarantine zone doing nothing	REF	REF	REF	REF
Being in quarantine to care for the COVID-19 infected patient				
Being in quarantine at home	2.11 (0.74–5.94)	2.20 (0.87–5.57)		2.28 (1.01–5.16) *
Having ended the duration in quarantine and normally work			2.14 (0.68–6.70)	
Normal working				
Duration participating in COVID-19 control (weeks)				
<1 week			REF	REF
1–2 week(s)				
2–4 weeks			0.28 (0.09–0.89) *	
4–8 weeks				
>8 weeks				0.31 (0.06–1.56)
Working directly in contact with COVID-19 patients frequently				
No			REF	REF
Yes			3.39 (1.17–9.86) *	1.77 (0.93–3.37)
Level of preparation before participating in COVID-19 control				
None	REF		REF	REF
Average			2.25 (0.78–6.48)	1.58 (0.86–2.92)
Adequate	0.51 (0.20–1.27)			
Level of equipment in current workplace conditions				
None		REF		REF
Average		0.19 (0.05–0.69) *		0.45 (0.14–1.45)
Adequate		0.12 (0.03–0.47) **		
Affected by workplace conditions				
No				REF
Yes				
Affected a lot by the community				
No	REF		REF	REF
Yes	5.39 (1.70–17.11) **		4.26 (0.91–19.95)	
Feeling stressed about current works				
No	REF	REF		REF
Yes	2.66 (0.98–7.25)	2.47 (0.99–6.17)		4.84 (2.40–9.75) ***
Feeling anxious about current works				
No		REF		REF
Yes		2.52 (1.07–5.92) *		
Feeling sad about current works				
No	REF	REF	REF	REF
Yes	1.91 (0.80–4.58)	3.71 (1.72–7.98) **	2.28 (0.86–6.00)	3.24 (1.76–6.00) ***

OR: odds ratio; CI: confidence interval; REF: reference; *, **, ***: significant at 0.05, 0.01, and 0.001.

## Data Availability

The data used to support the findings of this study are available from the corresponding author upon request.
